# Cost Evaluation of Minimally Invasive Tissue Sampling (MITS) Implementation in Low- and Middle-Income Countries

**DOI:** 10.1093/cid/ciab828

**Published:** 2021-12-15

**Authors:** Laura T R Morrison, Elizabeth G Brown, Christina R Paganelli, Suraj Bhattarai, Rahell Hailu, Gervais Ntakirutimana, Djibril Mbarushimana, Nuwadatta Subedi, Norman Goco

**Affiliations:** 1 RTI International, Durham, North Carolina, USA; 2 Gandaki Medical College Teaching Hospital and Research Center, Pokhara, Nepal; 3 College of Health Sciences, Addis Ababa University, Addis Ababa, Ethiopia; 4 Kigali University Teaching Hospital, Kigali, Rwanda; 5 University Teaching Hospital of Butare, Huye, Rwanda

**Keywords:** costs, LMICs, MITS, mortality surveillance

## Abstract

**Background:**

Low- and middle-income countries (LMICs) face disproportionately high mortality rates, yet the causes of death in LMICs are not robustly understood, limiting the effectiveness of interventions to reduce mortality. Minimally invasive tissue sampling (MITS) is a standardized postmortem examination method that holds promise for use in LMICs, where other approaches for determining cause of death are too costly or unacceptable. This study documents the costs associated with implementing the MITS procedure in LMICs from the healthcare provider perspective and aims to inform resource allocation decisions by public health decisionmakers.

**Methods:**

We surveyed 4 sites in LMICs across Sub-Saharan Africa and South Asia with experience conducting MITS. Using a bottom-up costing approach, we collected direct costs of resources (labor and materials) to conduct MITS and the pre-implementation costs required to initiate MITS.

**Results:**

Initial investments range widely yet represent a substantial cost to implement MITS and are determined by the existing infrastructure and needs of a site. The costs to conduct a single case range between $609 and $1028 per case and are driven by labor, sample testing, and MITS supplies costs.

**Conclusions:**

Variation in each site’s use of staff roles and testing protocols suggests sites conducting MITS may adapt use of resources based on available expertise, equipment, and surveillance objectives. This study is a first step toward necessary examinations of cost-effectiveness, which may provide insight into cost optimization and economic justification for the expansion of MITS.

The vast majority of global childhood deaths are disproportionately concentrated in low- and middle-income countries (LMICs) [1] and crafting meaningful interventions to reduce this mortality is contingent upon robust, cause-specific mortality data [2]. However, only about half of global deaths are attributed to a reliable cause [3]. In LMICs, cause of death—from stillbirths and neonates to adults—is often determined by reported or clinical symptoms, which limit the accuracy of cause of death determination and can significantly bias population-level mortality measures. Minimally invasive tissue sampling (MITS) is a standardized procedure using needle-drawn biopsies that has been shown to perform similarly well to conventional autopsy [4–6], and holds promise to improve the accuracy of cause of death information where conventional postmortem methods are not feasible, unacceptable, and costly [7–9]. MITS also holds the potential to improve other tools for measuring mortality, such as verbal autopsy, and improve the epidemiological knowledge base in concert with these surveillance tools [10].

Evidence documenting the validity [4, 5, 11, 12], acceptability [8, 11], and feasibility [13, 14] of MITS supports the procedure’s expanded use in LMICs, yet comprehensive estimates of the costs and cost-effectiveness to implement the procedure are needed [6]. Cost analyses are an important initial step in determining cost effectiveness against existing practices and informing budget decisions based on realistic implementation scenarios [15].

To date, no comprehensive estimates of the costs to implement MITS in LMIC settings have been published, leading to calls for more evidence [6]. The majority of studies examining costs associated with MITS or minimally invasive autopsy are conducted in high-income countries like the United States, United Kingdom, or European Union [16–18]. These studies hold less relevance for LMIC settings, as they capture costs associated with computerized tomography (CT) or magnetic resonance imaging (MRI) equipment to guide MITS, which are not widely accessible in LMIC clinical facilities, and which are not contemplated in current protocols for MITS in LMICs.

Several studies reference costs to conduct MITS in LMIC settings but do not conduct a comprehensive accounting of the costs. An examination of barriers to MITS implementation in Mozambique noted that complex microbiology platforms, the need for specialized labor, and the high cost of specific supplies like biopsy needles all present challenges to MITS expansion [19]. Feroz et al [20] found that healthcare workers in Pakistan perceived the initial investment cost of MITS to be high, which led to the belief that public investment was a necessity [20].

In this work, we aim to characterize the costs and cost drivers associated with conducting MITS in LMIC settings, by examining 4 sites where MITS has been implemented at pilot scale. As a result, this case study approach speaks to the potential costs of MITS, rather than generalizable findings. We capture costs from the perspective of the health care provider or health system in order to provide national health ministries and agencies with information on what MITS implementation may cost in order to inform efficient resource allocation decisions.

## METHODS

This study adopts a micro-costing approach to capturing costs associated with MITS from the healthcare provider perspective. “Bottom-up” micro-costing estimates the unit resources (eg, labor hours, materials) at the detailed activity level, and aggregates them to form summary totals [21]. This approach has several advantages: first, it is considered the most accurate cost estimation strategy for healthcare activities [22]; second, it enabled us to capture greater detail related to the resource requirements and cost drivers for MITS, which is useful for future applications in different contexts and future economic modeling.

We adopt the healthcare provider perspective because in LMICs, as in many countries, mortality surveillance activities are primarily conducted by healthcare providers. Providers’ activities are directed and funded by national heath ministries, public agencies, or other nonprofit entities interested in efficient resource allocation. For this reason, we acknowledge but do not include costs from the family member or societal perspectives, which often entails unexpected and often large costs to the family of the deceased.

## DATA COLLECTION

In consultation with subject matter experts, we identified key activities required to conduct MITS in a facility (eg, hospital, clinic, morgue) and in the community, and designed a survey to capture the associated unit-level direct costs that were incurred (Table 1). Direct costs were inclusive of all staff labor and materials costs, including administrative requirements, but did not include indirect costs like staff benefits or pre-existing infrastructure costs. We piloted the instrument in two sites before finalizing the survey instrument.

**Table 1.** Costs Information Collected From MITS Sites

Recurring Costs (per Case)					
Screening and Enrollment	Sample Collection	Processing	Analysis	Reporting	Cause of Death Determination
• Labor • Consumable materials	• Labor • Consumable materials by sampling technique • Number and type of samples collected • Staff transportation (if conducted in community)	• Labor • Cost by test type • Number and type of test conducted • Consumable materials • Testing fee and transport (if conducted off-site)	• Labor • Consumable materials	• Labor • Consumable materials	• Labor • Consumable materials
Initial investment					
Start-up costs				Capital costs	
• Ethics approval (labor and fees) • Protocol development (labor) • Data management system development (labor) • Community sensitization activities (labor)		• Initial training (labor, space, materials, other expenses) • Training additional staff (labor, space, materials, other expenses)		• Lab equipment • Office furniture • Technology (eg, computers, cameras) • Renovation	

We surveyed 4 sites conducting MITS at university hospitals in LMICs across Sub-Saharan Africa and South Asia. All sites were grantees of the MITS Surveillance Alliance Secretariat that received incentive grants to expand the use of MITS. Participating sites had experience conducting MITS (>10 MITS cases completed at the time of data collection) and represented varied case populations. Table 2 provides an overview of each site’s characteristics.

**Table 2.** Site Characteristics

	Site 1	Site 2	Site 3	Site 4
MITS case target population	6% infants 94% adults	100% adults	5% neonates 5% stillbirths 15% infants 75% adults	100% neonates
Number of samples taken per case (average case)	7	9	9	6
Type of samples collected (average case)	Brain, lung, liver, blood, CSF, marrow, lesions	Brain, lung, liver, blood, CSF, skin, pleural effusion, lesions, cancerous masses	Brain, lung, liver, blood, CSF, placenta, skin, lesions, cancerous masses	Brain, lung, liver, placenta, lesions, abdominal organs

Abbreviations: CSF, cerebral spinal fluid; MITS, minimally invasive tissue sampling.

We collected data in LMICs between February and August of 2020, before and during the early stages of the coronavirus disease 2019 (COVID-19) pandemic. At each site, the principal investigator and an employee knowledgeable of the site’s finances responded to the computer-based survey. The teams provided additional details and clarifications by follow-up teleconference and email. Each team reviewed and approved the final cost estimates. At sites where data were collected after COVID’s onset, we asked them to report the costs incurred prior to the pandemic, which most readily had affected the price of personal protective equipment.

Respondents provided resource information for activities associated with preparing the site to conduct MITS and conducting the MITS procedure, based on actual costs incurred rather than budgets or projected expenses (Table 1). This included activity-specific labor and materials associated with the MITS procedure and background information on MITS at their site (eg, case populations, context). Respondents recorded activity-specific materials consumed and labor hours by staff role for an average single MITS case. Per case or annual consumable expenses were defined as those that are used on an ongoing basis to support MITS and that are “used up” and replaced, including lab supplies and fees, reagents, storage rental, lab or office space, and office supplies. Nonconsumable expenses included costs that do not regularly recur over the course of conducting a MITS program, which we define as capital costs. For all fields, respondents were able to enter additional resources, staff, and activities.

To capture labor expenses, respondents entered all staff roles that typically contributed to the preparation or performance of MITS. For every staff role, teams indicated the number of persons performing the role, their compensation, and their typical hours worked per week at their site. Respondents provided the number of hours that staff members typically spent on a given activity for an average MITS case. Respondents recorded these hours separately from time spent on administrative tasks related to MITS, like grant management, quarterly reporting, and equipment maintenance, for which we do not account in this study. We compared role-specific wage estimates given by sites to Serje et al’s estimates of health worker earnings as multiples of gross domestic product (GDP) per capita in low- and lower-middle income countries [23], finding all site estimates to be generally in line with these estimates, excepting site 2, which had higher labor rates, potentially due to its urban location.

## COST MODEL

We developed an Excel-based cost model to aggregate each sites’ activity-specific resource cost estimates and categorized them into 1-time, initial costs (start-up and capital costs) required to be in place for a MITS program to begin and recurring per-case and annual costs to conduct MITS at each site. Costs provided in local currencies were converted to US dollars. We provide a financial accounting of these costs —that is, we did not adjust for depreciation and presented the purchase cost for these items that had been purchased recently or through the MITS Surveillance Alliance grant. We excluded costs to perform additional research activities.

The majority of cost categories required simple aggregation. Staff compensation varied by site, including with regular salaries or hourly wages, fixed incentive payments per-case, and per-diem payments for specific activities. We calculated activity-specific labor costs by activity, based on each site’s reported compensation schedule. Where staff were paid hourly wages, we multiplied their wage by the number of hours they spent on an activity, whereas for salaried staff we estimated an hourly wage where appropriate. Start-up costs included some nonconsumable expenditures like equipment purchases and facility renovations that were not used exclusively for MITS. In these cases, we adjusted these costs by the utilization rate of the MITS program. Site-specific estimates were reviewed with respondents before finalizing results.

## RESULTS

### Recurring Per-Case Costs

Table 3 presents cost results from the 4 sites implementing MITS included in our study, organized by recurring costs and 1-time, start-up costs. The cost to conduct a single MITS case ranged between $609 and $1028 and was driven by 3 main factors: labor, testing expenses, and MITS kits (ie, standardized kits containing materials provided by the MITS Surveillance Alliance to conduct procedure).

**Table 3.** Summary of Costs to Implement MITS, by Site (USD)

	Site 1	Site 2	Site 3	Site 4
Recurrent costs				
MITS procedure (total/case)	$889	$1028	$906	$609
Labor	$211	$386	$314	$165
Testing	$261	$228	$161	$11
Test transport			$5	
Materials	$19	$17	$28	$35
MITS kits	$398	$398	$398	$398
One-time costs				
Total	$37 434	$21 563	$44 678	$6784
Capital costs (total)	$22 551	$6535	$36 505	$2050
Lab equipment	$8469	$500	$6235	$2050
Office furniture	$1538	$1501		
Technology	$4903	$1533	$3351	
Renovation	$7640	$3001	$26 919	
Start-up costs (total)	$14 884	$15 027	$8173	$4734
Training (total)	$3149	$4146	$7343	$2672
Training (labor)	$520	$1267	$2900	$457
Training (materials, space + MITS kits)	$2629	$2065	$3643	$1922
Training additional staff		$814	$800	$293
Implementation preparation (total)	$11 735	$10 881	$830	$2062
Ethics approval	$378	$1898	$160	$263
Protocol development	$756	$4377	$400	$1512
Data management system	$10 475	$500	$100	$175
Community sensitization	$126	$876	$170	$112
Misc expenses and activities		$3231		

Abbreviation: MITS, minimally invasive tissue sampling.

Labor costs constitute roughly 25% of MITS costs per case. Although regional wage differences explain some differences across sites, sites reported a high degree of heterogeneity in staff roles participating in an average MITS procedure (Table 4). All sites had at least 2 pathologists engaged and in 1 case, up to 5. At least 1, but often 2 laboratory technicians supported the procedure. A multitude of additional other staff were engaged (between 6 and 11 individuals). In many cases, nonpathologist and laboratory technician staff roles were engaged at specific parts of the MITS. Staff were paid either a flat amount per case or paid regular wages for their time.

**Table 4.** Testing Costs by Site, Average per Case

	Site 1		Site 2		Site 3		Site 4	
Testing Cost per Case	Total Cost/ Case	No. Tests/Case	Total Cost/ Case	No. Tests/ Case	Total Cost/ Case	No. Tests/ Case	Total Cost/ Case	No. Tests/ Case
Histology	$50.51	5.0	$91.70	5.0	$42.00	4.0	$10.00	1.0
Microbiology	$33.67	5.0	$50.02	6.0	$28.00	4.0	…	…
IHC	…	…	-	…	$58.80	1.2	…	…
Biochemistry/serology	…	…	$75.03	9.0	…	…	…	…
Haematology	…	…	$6.67	2.0	…	…	…	…
Special stains	$176.66	17.0	$4.38	0.2^a^	$32.40	3.0	$0.70	0.1^b^
Total	$260.84	27.0	$227.80	22.2	$161.20	12.2	$10.70	1.1

Abbreviation: IHC, immunohistochemistry.

^a^Site reported conducting test in one of every t cases.

^b^Site reported conducting test in 1 of every 10 cases.

Costs to conduct sample analysis, or testing, make up a significant portion (18%) of the per case cost and are determined by the type and number of tests conducted per case, which varied by the site-specific protocols (Table 5). All sites conducted at least 1 histology test per case, although many conducted over 4 per case. Many sites conducted microbiological and special stains tests. No sites reported using molecular, cytology, TaqMan® array card (TAC) polymerase chain reaction (PCR), or traditional PCR testing. Although test costs varied, the sites reflect a consensus that immunohistochemistry (IHC), special stains, and histology were most costly.

**Table 5.** Staff Contributing to MITS Procedure by Site, Average Case

	Site 1		Site 2		Site 3		Site 4	
Staff Position	Number of Staff	Hours per Case^a^	Number of Staff	Hours per Case^a^	Number of Staff	Hours per Case^a^	Number of Staff	Hours per Case^a^
Pathologists	2	1.5	3	4.5 ^b^	2	4	5	5.4 ^c^
Lab techs	2	2.5	2	3.25 ^d^	2	5	1	4
Social scientists	…	…	1	4	1	2	…	…
Microbiologists	…	…	1	3.5	1	4	…	…
Administrative/ logistics	Security logistician ^e^ Accountant ^e^	0.5 … …	Assistant data enumerator ^e^	3 …	…	…	Site supervisor Grant manager ^e^	2 …
Other staff	Clinical specialist MITS specialist MITS assistant Nurse counselor	0.5 1 1 0.5	Physician	0.5	…	…	Pediatrician	4
Total	9	11.5	9	32	6	24	8	37

Abbreviation: MITS, minimally invasive tissue sampling.

^a^Hours per case denotes hours per individual employee (eg, at site 1: 2 pathologists spent 1.5 hours each, totaling 3 hours per case.)

^b^Pathologists 1, 2 and 3 spend 7, 3, and 3.5 hours on a typical case, respectively.

^c^One lead pathologist spends 11 hours per case, and 4 supporting pathologists spend 4 hours per case.

^d^One lab tech spends 2.5 hours, and the other spends 5 on a typical case.

^e^Denotes staff roles that did not record an hourly contribution to MITS procedure but are included in labor cost per case.

MITS kits, which are provided to sites with the needed supplies to conduct the MITS procedure, comprise the largest portion (48%) of the per case MITS cost. This cost includes a standard battery of supplies ($225), a back-up kit sent with each batch of 10 kits ($29 per kit), and assembly and shipping costs to send kits from the United States ($142 labor and shipping). Sites received these kits directly from the MITS Surveillance Alliance program.

### Initial Costs (One-Time)

The 1-time costs required to establish a MITS program, divided into start-up activity and capital costs, far outweigh the cost to complete a single case and differ substantially by site. The wide variation in capital costs by site is driven primarily by the preexisting infrastructure available. Renovation expenses, where necessary, drove capital costs, as some sites adapted existing lab and office space to accommodate MITS. Other capital expenses included equipment and machinery. Site 1 was least well-resourced initially and required a costly autopsy table and embedding machine, whereas site 4 had fewer needs, investing only in a microscope.

Start-up costs varied notably by site and reflect each site’s investment in specific pre-implementation activities. For instance, the volume of staff trained in each site differed, which reflects a diversity of available staff roles and/or those selected to receive MITS training. Guidelines shared by the MITS Surveillance Alliance did not stipulate a specific number of hours to complete pre-implementation activities yet did provide standards for their completion.

## Discussion

The purpose of this cost analysis was to understand the cost implications of MITS implementation in a range of different LMIC contexts, providing a range of transparent cost estimates that reflect the resources needed for expanding MITS in new locations. These cost estimates are an initial milestone in demonstrating the comprehensive costs of conducting MITS incurred by a provider. Our findings identify important cost drivers and opportunities for reducing cost.

The lack of standardization across sites in terms of labor and testing—key drivers of incremental costs—suggests that staff roles and testing protocols may be varied without sacrificing the accuracy of cause of death findings. This variation points to the potential flexibility around labor and testing to reduce per case costs and adapt MITS protocols around resource availability or surveillance goals. First, the notable variation in staff roles applied by each site has the potential to both give greater flexibility to sites that do not have a large number of specialty clinicians available and points to opportunities to reduce labor costs. For instance, with a core team of pathologists for supervision, other staff roles could perform some tasks in place of pathologists. Forthcoming research related to “task-shifting” across roles to conduct MITS explores this in-depth and is an important area of future study.

Second, the varied type and number of tests conducted across the 4 sites suggests that improved insight into causes of death from MITS can still be achieved through nonuniform sample testing protocols. Each site’s protocols reflect site-specific surveillance objectives, testing budgets, and the equipment and expertise available. In our study, sites used standard testing procedures across the sites, but no site used more advanced tests, like TAC PCR and traditional PCR tests. This may be due to the limited availability of testing equipment, such as PCR machines, or because more tests may not imply improved cause of death information. Due to the initial investment in infrastructure to conduct tests using molecular techniques like PCR, their inclusion would likely increase the cost to perform MITS, despite the decreasing costs of PCR, in part due to widespread PCR testing for COVID-19. A growing body of evidence suggests that incremental testing may increase data associated with the presence of pathogens yet may not necessarily increase the precision and accuracy of cause of death determination [24]. Ritter et al similarly explore the use of histopathology in MITS in this supplement. This notion raises the importance of future study around optimizing test selection for cost-effectiveness to improve mortality surveillance.

As MITS is conducted at relatively few locations globally, expanding its use gives opportunity to reduce per case costs by procuring supplies locally. Although the costs associated with MITS kits are substantial—between 35% and 65% of per case costs—they reflect MITS implementation at pilot-scale and could be reduced in the future by local procurement. The contents of the kits are not highly specialized, suggesting that they may not be limited by supply chains in LMICs.

We find that incremental (per case) costs to conduct MITS were relatively minor, once the initial investments to perform MITS were made. This is positive, in that low incremental costs may avoid the challenges of disinvestment over time, as many public health funders may be wary of securing substantial long-term resources to sustain highly costly programs.

The initial investments varied by site due to the differing needs and existing infrastructure for capital goods to conduct MITS and highlights the importance of evaluating the infrastructure in-place when considering costs to conduct MITS at a new site. Where necessary, renovation of office and lab space comprised a large portion of these costs, as substantial space is needed to conduct MITS. However, no capital expense was required universally by site, suggesting that potential locations may have the majority of necessary items on hand. Activities to prepare for MITS implementation, including training, reflected each site’s ability to devote resources and/or priority given to these activities.

### Limitations

This study represents a small number of sites, and although each site’s costs and context vary, they illustrate the diversity in approaches to implement MITS. As such, obtaining precisely comparable information from each site was difficult. Although we conduct limited cross-site comparisons in this analysis, the individual differences of the economies and healthcare environments limit the utility of these comparisons. Comparing specific resources or staffing roles may be most telling for contextualizing these findings in a new location. We also do not discuss the effectiveness of the MITS program. In the future, it will be important to weigh the body of evidence for MITS to assess the value in replacing existing routine surveillance systems.

Our study did not include all possible costs of MITS implementation, such as family counseling, socio-anthropological support (eg, rumor tracking), and clinical data abstraction. Importantly, our study also did not include program administration costs (eg, grants management, reporting, internal and external communications), which are a fundamental component of MITS program operational expenses. We were, however, careful to exclude them from our estimates. Administrative costs are notoriously challenging to include and interpret, and often favor a “top-down” costing methodology to account for administrative burden, which is often less precisely tracked and can be under-reported in “bottom-up” costing [21]. Furthermore, if collected at pilot scale, these costs can heavily differ from those of an expanded program, which may be integrated into existing administrative systems or more complex set of staff and activities.

Finally, all activities and costs represented in this study are defined by external grant funding and the technology available to each site. Thus, these results may not represent the activities that would be conducted in the absence of a budget constraint or at expanded scale, but do represent illustrative examples of MITS implementation in LMIC settings where these constraints are common. For instance, sites implementing MITS beyond a pilot-scale may increase community sensitization work in order to improve MITS’ acceptability and overall success. The scale and funding of the costs represented could be both higher and lower than the costs of self-funded MITS implementation.

## CONCLUSION

This study is a first step toward additional necessary examinations of cost considerations, including cost-comparison and cost-effectiveness studies, which further provide needed economic justification for the expansion of MITS for improving cause of death data.

## Notes

***Acknowledgments.*** We are grateful to the sites who reported costing data for this study and to the employees of the sites performing this work; without their dedication, this study would not be possible.

***Supplement sponsorship.*** This supplement is sponsored by MITS Surveillance Alliance Secretariat, led by RTI International, with funding from the Bill and Melinda Gates Foundation.

***Financial sources.*** This work was supported by the MITS Surveillance Alliance. This work was supported, in whole or in part, by the Bill & Melinda Gates Foundation (grant number OPP1180554). Under the grant conditions of the Foundation, a Creative Commons Attribution 4.0 Generic License has already been assigned to the Author Accepted Manuscript version that might arise from this submission.

***Potential conflicts of interest.*** The authors: No reported conflicts of interest. All authors have submitted the ICMJE Form for Disclosure of Potential Conflicts of Interest. Conflicts that the editors consider relevant to the content of the manuscript have been disclosed.
